# The Impact of a Multidisciplinary Team Conference on Non-Small Cell Lung Cancer Care: Time Barriers and Long-Term Outcomes

**DOI:** 10.3390/jcm13175276

**Published:** 2024-09-05

**Authors:** Somcharoen Saeteng, Busyamas Chewaskulyong, Chaiyut Charoentum, Nirush Lertprasertsuke, Juntima Euathrongchit, Pattraporn Tajarernmuang, Pitchayaponne Klunklin, Sophon Siwachat, Sarawut Kongkarnka, Yutthaphan Wannasopha, Thatthan Suksombooncharoen, Thanika Ketpueak, Apichat Tantraworasin

**Affiliations:** 1 Clinical Surgical Research Center, Chiang Mai University, Chiang Mai 50200, Thailand; tengearneae@gmail.com (S.S.); phonsiwachat@hotmail.com (S.S.); 2 Department of Surgery, Faculty of Medicine, Chiang Mai University, Chiang Mai 50200, Thailand; 3 Department of Internal Medicine, Faculty of Medicine, Chiang Mai University, Chiang Mai 50200, Thailand; chaiyut.charoentum@cmu.ac.th (C.C.); pattraporn.t@cmu.ac.th (P.T.); thatthan.s@cmu.ac.th (T.S.); thanika.k@cmu.ac.th (T.K.); 4 Department of Pathology, Faculty of Medicine, Chiang Mai University, Chiang Mai 50200, Thailand; nlertpra@hotmail.com (N.L.); sarawut.kon@cmu.ac.th (S.K.); 5 Department of Radiology, Faculty of Medicine, Chiang Mai University, Chiang Mai 50200, Thailand; juntima.eua@cmu.ac.th (J.E.); pitchayaponne.kl@cmu.ac.th (P.K.); yutthaphan.w@cmu.ac.th (Y.W.); 6 Clinical Epidemiology and Clinical Statistics Center, Faculty of Medicine, Chiang Mai University, Chiang Mai 50200, Thailand

**Keywords:** MDT, NSCLC, time barrier, lung cancer care, waiting time, inverse-probability weighting propensity score

## Abstract

**Background/Objectives**: The prolonged time to reach investigation and management decisions in non-small cell lung cancer (NSCLC) patients can negatively impact long-term outcomes. This retrospective cohort study aims to assess the impact of a multidisciplinary team conference (MDT) on NSCLC care quality and outcomes. **Methods**: This retrospective study included resectable NSCLC patients who underwent pulmonary resection at Chiang Mai University Hospital, Thailand, from 1 January 2009 to 31 December 2021. Patients were divided into two groups: non-MDT and MDT groups, based on the initiation of MDT on 1 March 2018. The study compared overall survival, disease-free survival, and waiting times for investigation and surgery between the two groups. The effect of MDT on these outcomes was analyzed using multivariable analysis with inverse-probability weighting propensity scores. **Results**: The study included 859 patients, with 583 in the non-MDT group and 276 in the MDT group. MDT groups had a higher proportion of stage I and II NSCLC patients undergoing pulmonary resection (78.6% vs. 59.69%, *p* < 0.001). In multivariable analysis, patients in the MDT group had a significantly higher likelihood of longer survival compared to the non-MDT group (adjusted HR 0.23, 95% CI 0.09–0.55). Median waiting times for bronchoscopy (3 days vs. 12 days, *p* = 0.012), pathologic report (7 days vs. 13 days, *p* < 0.001), and surgery scheduling (18 days vs. 25 days, *p* = 0.001) were significantly shorter in the MDT group. **Conclusions**: An MDT has a survival benefit in NSCLC care and improves waiting times for investigation and treatment steps. Further studies are needed to validate these results.

## 1. Introduction

Lung cancer is the leading cause of cancer-related deaths in the United States and many countries worldwide. It accounts for 14% of all cancer diagnoses but is responsible for 25% of all cancer deaths, surpassing the combined mortality rates of breast, colorectal, pancreas, and prostate cancers [1]. Non-small cell lung carcinoma (NSCLC) represents 85% of lung cancer cases [2]. The average 5-year overall survival rate ranges from 12 to 90%, depending on the stage of the disease [3]. Early-stage lung cancer is associated with longer overall survival. Accurate diagnostic work-up involves procedures such as transthoracic needle aspiration, core needle biopsies, bronchoscopic biopsy (with or without endobronchial ultrasound-guide biopsy—EBUS), and obtaining adequate tissue for molecular subtyping. Computed Tomography (CT) scans and Positron Emission Tomography (PET) scans are used for staging and to guide treatment decisions. Treatment options for lung cancer include surgery, radiotherapy, chemotherapy, targeted therapies, immunotherapy, palliative care, or a combination of these approaches [4,5]. Multiple clinicians are typically involved at each step of the management process, and the time taken to reach a treatment decision can be prolonged. Unfortunately, delays at each step of investigation can lead to delayed diagnosis and treatment. Numerous previous studies have reported that longer intervals between the first visit and the initiation of treatment are associated with worse survival rates [6,7]. To address this issue, a multidisciplinary team (MDT) approach has been initiated, utilizing evidence-based management to improve treatment access and enhance coordination and communication among healthcare professionals [8,9,10,11]. The benefits of MDT involvement include greater accuracy and completeness in diagnosis and staging [12,13,14], improved adherence to therapeutic guidelines, and earlier initiation of treatment [15].

Consequently, MDT plays a pivotal role in optimizing outcomes for lung cancer [16,17,18,19]. To close the practice gap in diagnosis and treatment, we have implemented an MDT conference for thoracic oncology at our institute since March 2018. Our team consists of thoracic surgeons, medical and radiation oncologists, pulmonologists, radiologists, pathologists, as well as residents or fellows in each specialty. Other specialties will be invited if they become involved. The MDT conference at our center occurs once a week and lasts for two hours. Due to time constraints and the complexity of cases, we limit the number of patients presented to 5 to 15. The MDT is led by surgeons, oncologists, and pulmonologists, and the majority of patients discussed are referrals from thoracic surgeons. However, any attending physician at our center who encounters patients with lung cancer can refer them for discussion. The selection of patients for presentation is based on the attending physician’s assessment that multidisciplinary input may be beneficial in confirming the diagnosis or recommending appropriate therapy. By bringing together experts from various specialties, our MDT aims to provide comprehensive and collaborative care to improve patient outcomes. The objective of this study is to investigate the influence of an MDT conference on lung cancer care at Chiang Mai University Hospital. Specifically, we aim to assess the impact of the MDT conference on the time barriers associated with lung cancer diagnosis and treatment. Additionally, we seek to explore the effects of the MDT conference on tumor recurrence and overall survival in patients with resectable NSCLC at Chiang Mai University Hospital.

## 2. Materials and Methods

### 2.1. Patients and Study Design

A non-randomized therapeutic research design was employed for this retrospective data collection, observational cohort study. The study included all adult patients (age > 18 years old) with a confirmed histologic diagnosis of NSCLC who underwent curative pulmonary resection with systematic lymph node dissection or sampling at the Department of Surgery, Faculty of Medicine, Chiang Mai University, Chiang Mai, Thailand, between 1 January 2009 and 31 December 2021. All data were extracted from the data recording system. Patients with incomplete outcome variables were excluded from the study. We obtained approval from the Institutional Review Board (Research Ethics Committee Faculty of Medicine, Chiang Mai University, Research ID 9053/Study Code: SUR-2565-09053), approval number 218/2022), with a waiver for written informed consent due to the retrospective nature of the study. Data were accessed from the medical records system for research purposes between 1 July 2022 and 30 March 2023.

**Figure 1:**
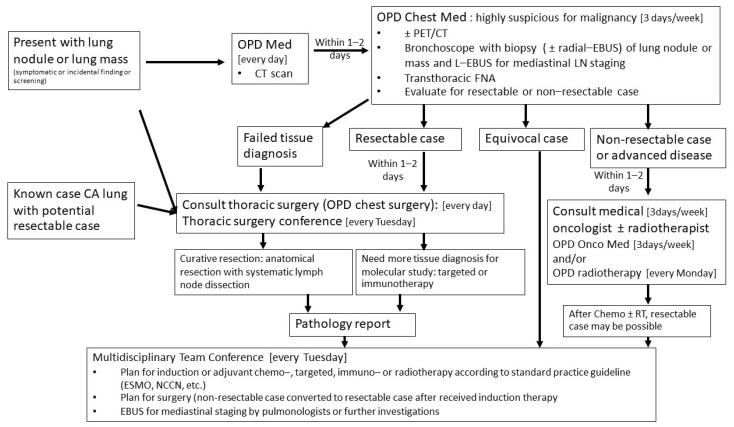
A demonstration of the framework for the multidisciplinary team approach in patients presenting with lung mass, lung nodule, or lung cancer. (Abbreviations: OPD—Out-Patient Clinic, CT—Computed Tomography, PET/CT—Positron Emission Tomography/Computed Tomography, EBUS—Endobronchial Ultrasound, L-EBUS—Linear Probe EBUS, FNA—Fine Needle Aspiration, Onco Med—Oncology Medicine, Chemo—systemic treatments including chemotherapy, immunotherapy, and targeted therapy, RT—radiotherapy, ESMO—European Society for Medical Oncology, NCCN—National Comprehensive Cancer Network, CA—cancer, MED—medicine).

**Figure 2:**
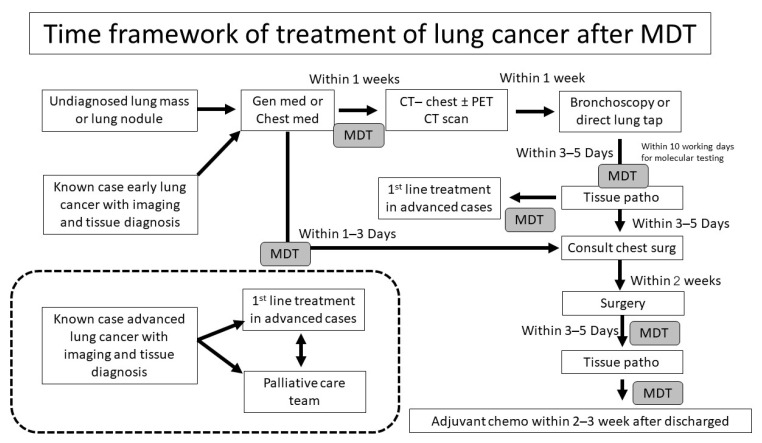
Demonstration of time framework of treatment for lung cancer after MDT conference. (Abbreviations: MDT—multidisciplinary team, gen med—general medicine, chest med—chest medicine (pulmonology medicine), CT—Computed Tomography, PET/CT—Positron Emission Tomography/Computed Tomography, tissue patho—tissue pathology, chest surg—chest surgery (thoracic surgery), chemo—chemotherapy.

The data extracted from the registry databases included patient characteristics, disease characteristics, pathological results, in-hospital mortality, follow-up data, and patient status at last follow-up. The timing for each step of investigation and treatment was collected, including the date at first visit, the date of bronchoscopy or intervention, the date of pathological reports, the date of consultation for chest surgery, the date of scheduling for surgery, the date of surgery, the date of discharge, the date of the last follow-up, and date of tumor recurrence or death. All patients were divided into two groups: the non-MDT group and the MDT group. The cut-off point for the time difference between the two groups was 1 March 2018, which marked the initiation of the MDT. The framework for the MDT approach for patients presenting with lung mass, lung nodule, or lung cancer is demonstrated in Figure 1, and the time framework for the treatment of lung cancer after the MDT conference is shown in Figure 2.

The MDT framework for managing patients with lung masses, lung nodules, or lung cancer encompasses all relevant outpatient clinics, including internal medicine, pulmonology, oncology, radiotherapy, and thoracic surgery. This framework was developed to streamline the often-time-consuming process of scheduling appointments across multiple outpatient clinics, ensuring that a patient can see multiple specialists within a short period if necessary.

For instance, if a patient visits the internal medicine outpatient department (OPD) on a Monday, they can be referred to the pulmonology OPD on the same day or within two days and then proceed to the thoracic surgery OPD within the same day (Figure 1). Additionally, we implemented a fast-track system for further investigations and treatments, as demonstrated in Figure 2.

The objective of this time-sensitive framework for lung cancer management is to initiate treatment as early as possible while ensuring that the maximum waiting times are not exceeded. It is well established that shorter intervals between diagnostic investigations and the initiation of definitive treatment lead to improved oncological outcomes for lung cancer patients. Therefore, the waiting period for a CT scan, bronchoscopy, or direct lung biopsy should be within one week, pathology reports should be available within 3–5 days, and surgery should be scheduled within two weeks. Systemic treatment should commence immediately once the patient’s performance status is deemed suitable. This framework emphasizes the importance of physician awareness throughout the entire process of investigation and treatment, with the goal of minimizing delays and initiating treatment as early as possible. However, the multidisciplinary assessment throughout each stage of investigation and treatment is tailored to the patient’s performance status and comorbidities. Comprehensive patient information and thorough discussions regarding disease prognosis are essential and should be provided to all patients and their relatives. This approach ensures that each patient receives the most optimal care possible.

### 2.2. Endpoints

The primary outcome assessed was the overall survival between the two groups. The survival time for each individual patient was calculated from the date of surgery to the date of death for patients who died, or to the date of the last contact for those who remained alive or were lost to follow-up. The secondary outcomes included the time (in days) between the first visit and bronchoscopic biopsy, the time between bronchoscopic biopsy and the pathological report, the time between the first meeting with the surgeon and the surgery date, in-hospital mortality, and tumor recurrence (the interval between the surgery date and the first documented tumor recurrence).

### 2.3. Sample Size Calculation and Statistical Analysis

In reference to the study of Gaudioso et al. [18], we calculated the sample size based on the mean survival time between the two groups (MDT versus non-MDT) using a test for two independent means. The ratio of MDT to non-MDT was 1:1, with an alpha value of 0.05 and a beta value of 0.8. The mean and standard deviation of the non-MDT group were 36.9 (30.2) and for the MDT group were 19.3 (15.8). The estimated sample size was 30 cases in each group. However, we utilized all the available data, totaling 859 cases, 583 cases in the non-MDT group and 276 cases in the MDT group, for data analysis. 

For data analysis, our initial plan was to analyze the data using propensity score-matched analysis to reduce the effects of confounding, minimize selection bias, and account for the differences in observed baseline patient and disease characteristics between the MDT and non-MDT groups. Logistic regression was used to calculate a propensity score, which evaluated confounding by indication and/or baseline covariates between the two groups, including stage of disease, tumor grading, age, Chalson comorbidity index (CCI), sex, smoking status, surgical procedures, and pathological characteristics. However, after preforming 1:1 propensity score matching, we found that the baseline patient and disease characteristics were still imbalanced, indicating that the matching method was not appropriate for this cohort. Therefore, we decided to perform an inverse-probability weighting propensity score to identify the treatment effect between the two groups. Multivariable analysis, under inverse-probability weighting propensity score adjustment for age, sex, CCI, stage of disease, procedures, histology, type of lymph node dissection, visceral pleural invasion, intratumoral lymphatic invasion, intratumoral vascular invasion, perineural invasion, and tumor necrosis, was conducted to explore the association between MDT and overall survival, tumor recurrence, and in-hospital mortality. An adjusted survival curve was used to demonstrate the survival between the two groups. For secondary outcomes, time variables were presented as the median with interquartile ranges and compared between the two groups using the Wilcoxon rank sum test. Multiple imputations (MI) with a multivariate normal equation were performed for any variables with at least 10% missing values [20]. Results of the MI analysis were then compared to the results from a complete-case analysis, and if there were no differences, the results from complete-case analysis would be reported. A *p*-value of less than 0.05 denoted statistical significance. We used STATA version 17.0 software (STATA Corporation, College Station, TX, USA) for the statistical analysis.

## 3. Results

A total of 1003 patients were diagnosed with NSCLC during this study period, with 583 patients classified in the non-MDT group and 420 in the MDT groups. In accordance with the study of Gaudioso et al. [18], management plan changes after MDT conference discussions were categorized into six items. In our study, 19.8% of patients had a change in choice of treatment modality, 45.1% had the creation of a plan in the absence of one, 9.3% underwent observation with serial CT scans, 1.6% had a change in surgical technique, 16.7% required further diagnostic testing, and 7.4% were recommended adjuvant therapy. Out of the 420 patients, only 276 patients underwent surgical resection. Finally, a total of 859 patients were included in the data analysis for primary and secondary outcomes.

**Figure 3:**
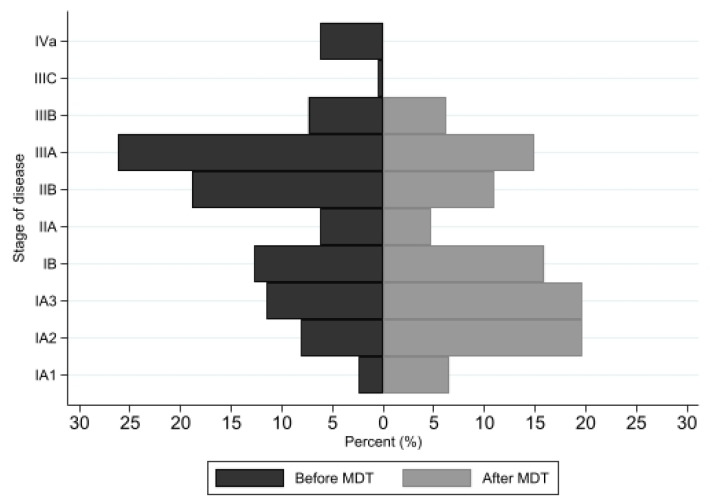
The proportion of patients in each stage of disease between the two groups (before MDT and after MDT initiation). (Abbreviations: MDT—multidisciplinary team).

The patient characteristics between the two groups are shown in Table 1. There were statistically significant differences in terms of insurance type, smoking status, history of cancer, Chlarson’s comorbidity index (CCI), stage of disease, histology, grading of cell differentiation, procedures, and type of lymph node dissection. Regarding the stage of disease, the proportion of early-stage disease in the MDT group was significantly higher than that in the non-MDT group (78.6% vs. 59.69%, *p* < 0.001), as shown in Figure 3.

**Figure 4:**
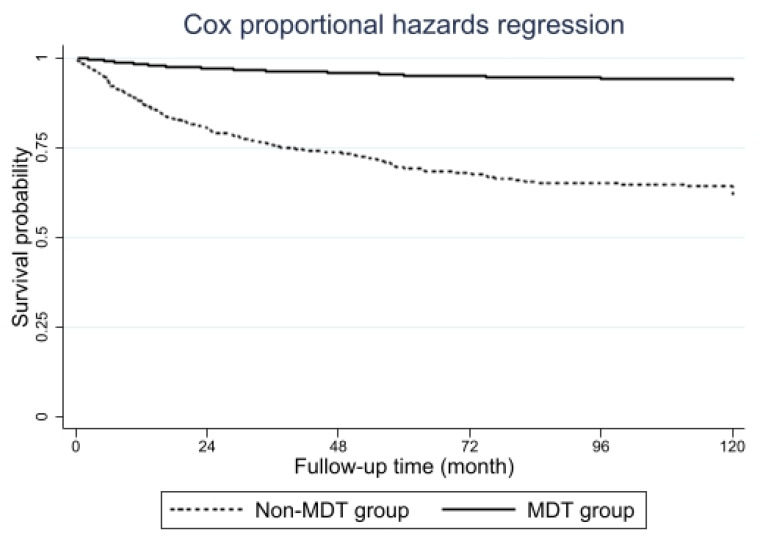
Adjusted survival curve between the two groups.

For univariable analysis, the proportion of overall mortality in the MDT group was significantly lower than that in the non-MDT group. Although the tumor recurrence rate in the MDT group was lower than that in the non-MDT group, the difference was not statistically significant, as shown in Table 2. In the multivariable analysis using inverse-probability weighting propensity score adjusted for age, sex, CCI, stage of disease, procedures, histology, type of lymph node dissection, visceral pleural invasion, intratumoral lymphatic invasion, intratumoral vascular invasion, perineural invasion, and tumor necrosis, patients in the MDT group were more likely to have longer survival compared to those in the non-MDT group (adjusted HR = 0.23, 95% CI 0.09–0.55), as shown in Table 3. The 3-year and 5-year overall survival rates in each stage of disease of patients in the MDT group were significantly longer than those of patients in the non-MDT group, as shown in Table 4. The adjusted survival curve between the two groups, analyzed by the multivariable Cox regression model, is demonstrated in Figure 4. However, there were no differences in the in-hospital mortality rate and tumor recurrence rate between the two groups.

**Figure 5:**
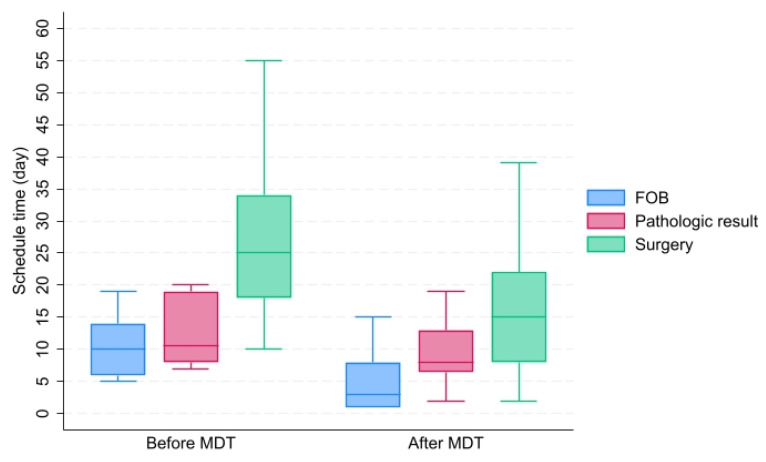
Box plot of the waiting time for bronchoscope (day), the waiting time for pathologic or cytologic results (day), and the schedule time for surgery (day) between the two groups, analyzed by Wilcoxon rank sum test. (Abbreviations: MDT—multidisciplinary team, FOB—fiberoptic bronchoscopy, pathologic result—the waiting time for pathologic or cytologic report, surgery—the waiting time for surgery).

For the secondary outcomes, the median waiting time for bronchoscopy (3 days, IQR = 1–8 versus 12 days, IQR = 6–14, *p* = 0.012), pathological report (7 days, IQR = 6–7 versus 13 days, IQR = 12–18, *p* < 0.001), and scheduling time for surgery (18 days, IQR = 7–32 versus 25 days, IQR = 12–34, *p* = 0.001) in the MDT groups were significantly shorter than those in the non-MDT group, as shown in Figure 5. The proportion of pathological reports received within 2 weeks after bronchoscopic biopsy was 94.3% in the MDT group compared to 62.9% in the non-MDT group, with *p* < 0001. Similarly, the proportion of waiting time for surgery within 4 weeks was 80.2% in the MDT group compared to 61.7% in the non-MDT group, with *p* < 0.001. Furthermore, the proportion of pathological reports received within 2 weeks after receiving a surgical specimen was 100% in the MDT group compared to 44% in the non-MDT group, with *p* < 0.001.

## 4. Discussion

MDT conferences for lung cancer care are crucial for optimizing patient outcomes, particularly in complex cases that require input from various specialists. Prior to implementing MDT initiatives, we observed that the overall survival of resectable NSCLC was not as high as expected. This realization prompted us to develop the MDT conference for lung cancer treatment discussions, not only for advanced-stage cases but also for early-stage cases. In the current era, pre-operative mediastinal staging using non-surgical approaches, such as endobronchial ultrasound-guided biopsy (EBUS), has become important. By incorporating highly experienced intervention pulmonologists and the availability of PET-CT scans, which are already reimbursed in our healthcare system since 2018, we aimed to enhance the MDT conference in lung cancer care. Our goal was to facilitate early diagnosis by reducing the time required to reach treatment decisions or to initiate further diagnostics, ultimately improving overall survival. 

Our study found that patients in the MDT group were more likely to have longer survival than those in the non-MDT group (adjusted HR = 0.23). The 3-year and 5-year survival rates were significantly improved in all stages of disease in the MDT group, consistent with the results reported by Gaudioso et al. [18]. A recent systematic review and meta-analysis demonstrated that MDT was protective of both 1-year survival (OR 3.23, 95% CI 2.85–3.68; *p* < 0.001) and overall survival (HR 0.63, 95% CI 0.55–0.72; *p* < 0.001) [13]. One of the reasons behind the improved survival rate is the enhancement of pre-operative mediastinal staging and the reduction in waiting time at each step of investigation and surgery. Our results demonstrate that the proportion of early-stage disease in the MDT group was significantly higher than that in the non-MDT group (78.6% versus 59.69%, *p* < 0.001). This suggests that, before the implementation of MDT conferences, patients with locally advanced or stage III disease who should have been treated with induction therapy were instead treated with surgical resection, potentially affecting their overall survival. On the other hand, in cases of locally advanced, recurrent, or advanced disease, obtaining further tissue diagnosis for molecular testing plays a vital role in targeted therapy and immunotherapy. The MDT conference helps reduce the time required for each process of investigation and treatment. Our study also found that the median waiting time for bronchoscopy, pathological reports, and scheduling surgery in the MDT groups was significantly shorter than that in the non-MDT group. These outcomes were observed after discussions in the MDT conferences, where we developed a fast-track system for investigations and set additional scheduled dates for lung cancer surgery. Following guidelines such as the British Thoracic Society Guidelines, the Canadian Society for Surgical Oncology guidelines, and the Canadian Association of Thoracic Surgery guidelines [21], we reach a consensus within the MDT group on waiting times for each step of investigation and treatment. This includes a waiting time of one week for performing a CT scan after the date of the request, a waiting time of 3–5 days for bronchoscopic biopsy, a waiting time of 7 days for tissue diagnosis results, and a waiting time of 10 days for molecular testing results. Additionally, the waiting time for surgery is less than 4 weeks. By reducing the waiting time at each step of investigation and treatment, we can potentially improve overall survival rates.

Previous studies have demonstrated the benefits of MDT conferences in facilitating discussions among different specialties involved in the diagnosis and treatment of lung cancer [22]. These conferences have been associated with shorter time intervals from initial consultations to treatment, higher proportions of mediastinal staging, lower proportions of unsuspected N2 disease, and higher adherence to clinical pathways, resulting in longer median overall survival in the MDT group [23,24,25,26]. Currently, MDT conferences and consensus play a vital role in all stages of lung cancer care, including in the preoperative and perioperative phase [27,28], patients suspected of lung cancer but unable to undergo tissue biopsies [29], palliative or end-of-life care settings [30,31], and treatment strategies, particularly for patients who are not suitable for surgical treatment in the early stages of disease [32]. MDT approaches aim to achieve the best quality care by involving patient-centered treatment decisions, supportive care, follow-up, and surveillance. An MDT facilitates effective communication by ensuring that all team members are familiar with the patient’s history and are involved in conceptualizing the treatment plan [24,33,34]. Although reaching 100% of planned waiting times may be challenging, we strive to develop appropriate solutions within the limited resources of our hospital.

The retrospective nature of this study is a limitation. The length of time difference between the two groups may have an impact on patient outcomes. Patient characteristics between the two groups differed, despite efforts to minimize this bias by using appropriate statistical analysis. We did not measure the time from first visit to the first treatment because the investigation and treatment processes vary for each patient, especially in cases where lung cancer diagnosis is equivocal. Therefore, determining the actual time from the first visit to the first treatment is challenging. Consequently, we opted to use waiting times for investigation and treatment as a proxy for the benefits of MDT conferences.

## 5. Conclusions

MDT conferences have demonstrated a survival benefit in NSCLC care and have an effect on the waiting time for each step of investigation and treatment. Our results confirm the existing evidence that suggests MDT facilitates the provision of high-quality lung cancer service, even in hospitals with limited resources. However, it is important to note that all the evidence is based on observational studies, and more quality studies are needed to confirm the association between MDT conferences and improvements in short- and long-term outcomes for lung cancer patients.

## Figures and Tables

**Table 1:** Patient characteristics between two groups.

Variables	Full Cohort (n = 859)			
	MDT n = 276	Non-MDT n = 583	*p*-Value	SMD
Age (year), mean ± SD	63.17 ± 10.29	62.40 ± 10.45	0.314	0.074
Gender, n (%)			0.121	0.116
Female	129 (46.74)	239 (40.99)		
Male	147 (53.26)	344 (59.01)		
Insurance type, n (%)			<0.001	0.605
UCS	75 (27.17)	311 (53.34)		
CSMBS	171 (61.96)	235 (40.31)		
SSS	15 (5.43)	33 (5.66)		
Others	15 (5.43)	4 (0.69)		
Smoking status, n (%)			<0.001	0.564
Non-smoking	143 (51.81)	148 (25.39)		
Smoking	133 (48.19)	435 (74.61)		
History of cancer, n (%)	54 (19.57)	36 (6.17)	<0.001	0.408
Chlarson’s comorbidity index, median (IQR)	3 (2–5)	2 (1–3)	<0.001	0.965
Stage of disease, n (%)			<0.001	0.653
I	170 (62.73)	202 (34.65)		
II	43 (15.87)	146 (25.04)		
III	58 (21.40)	199 (34.13)		
IV	0	36 (6.17)		
Histology, n (%)			0.022	0.206
Adenocarcinoma	189 (68.48)	372 (63.81)		
Squamous cell carcinoma	41 (14.86)			
others	46 (16.67)			
Cell differentiation, n (%)			<0.001	0.853
Good differentiation	124 (44.93)	173 (35.52)		
Moderate differentiation	65 (23.55)	198 (40.66)		
Poor differentiation	29 (10.51)	101 (20.74)		
Undifferentiated	2 0.72)	15 (3.08)		
Unknown	56 (20.29)	0		
Intratumoral vascular invasion, n (%)	95 (34.42)	224 (38.42)	0.290	0.083
Intratumoral lymphatic invasion, n (%)	207 (75.00)	421 (72.21)	0.411	0.063
Perineural invasion, n (%)	12 (4.35)	24 (4.12)	0.875	0.011
Tumor necrosis, n (%)	77 (27.90)	167 (28.64)	0.871	0.017
Procedure, n (%)			0.001	0.303
Wedge resection	74 (26.81)	96 (16.58)		
Segmentectomy	11 (3.99)	16 (2.76)		
Lobectomy	190 (68.84)	455 (78.58)		
Pneumonectomy	1 (0.36)	12 (2.07)		
Type of LN dissection, n (%)			<0.001	0.659
No LN dissection	47 (17.03)	0		
Systematic LN sampling	62 (22.46)	121 (20.75)		
Systematic LN dissection	167 (60.51)	462 (79.25)		
Propensity score, median (IQR)	0.13 (0.44–0.86)	0.01 (0.04–0.20)	<0.001	

CSMBS, Civil Servant Medical Benefit Scheme; SSS, Social Security Scheme; UCS, Universal Coverage Scheme; LN, lymph node; SMD, Standardized mean difference; IQR, interquartile range.

**Table 2:** Short-term and long-term results between the two groups.

Outcomes	Full Cohort (n = 859)		
	MDT n = 276	Non-MDT n = 583	*p*-Value
Length of hospital stay (day), median (IQR)	8 (5–12)	7 (5–10)	0.056
In-hospital mortality, n (%)	4 (1.45)	11 (1.89)	0.785
Tumor recurrence, n (%)	41 (14.86)	171 (30.50)	0.792
Time to recurrence (month) Median (IQR)	13.45 (4.15–20.52)	17.5 (8.97–39.70)	0.002
Overall mortality, n (%)	18 (6.52)	203 (35.43)	<0.001
Total follow-up time (month) Median (IQR)	120.23 (58.47–176)	106.87 (40–155.63)	0.007

**Table 3:** Treatment effects of outcome variables analyzed by multivariable regression analysis and inverse-probability weights propensity score analysis comparing MDT group versus non-MDT group.

Outcomes (MDT versus non-MDT)	Multivariable Regression Analysis		Inverse-Probability Weights Propensity Score Analysis	
	Estimates (95% CI)	*p*-Value	Estimates (95% CI)	*p*-Value
In-hospital mortality	0.80 (0.19–3.37) ^a^	0.760	1.01 (0.98–1.03)	0.518
Tumor recurrence	0.80 (0.50–1.28) ^b^	0.344	0.66 (0.33–1.36)	0.260
Overall mortality	0.17 (0.09–0.30) ^b^	<0.001	0.23 (0.09–0.55)	0.001

^a^ Analyzed by logistic regression analysis adjusted by age, sex, CCI, smoking status, stage of disease, and procedures, reported as odds ratio (95% confidence interval). ^b^ Analyzed by Cox regression analysis adjusted by age, sex, CCI, stage of disease, procedures, histology, type of lymph node dissection, visceral pleural invasion, intratumoral lymphatic invasion, intratumoral vascular invasion, perineural invasion, and tumor necrosis, reported as hazard ratio (95% confidence interval).

**Table 4:** The 3-year and 5-year overall survival in each stage of disease between the two groups.

Study Group	IA1	IA1	IA3	IB	IIA	IIB	IIIA	IIIB
3-year OS, (%)								
MDT	100	100	97.78	95.05	100	96.30	92.43	93.33
Non-MDT	92.86	87.23	89.23	80.56	77.78	77.98	67.11	64.29
5-year OS, (%)								
MDT	100	100	97.78	95.05	100	92.28	92.43	93.33
Non-MDT	85.71	82.98	86.15	76.39	75.00	68.81	62.42	61.90

OS = overall survival.

## Data Availability

Data cannot be shared publicly due to ethics committee law. Data are available on request and de-identified data sets are currently held by the Thoracic Surgery Unit of the Department of Surgery, Faculty of Medicine, Chiang Mai University, Chiang Mai, Thailand.
